# Supplementary material for: Enhancing Alginate Hydrogels as Possible Wound-Healing Patches: The Synergistic Impact of Reduced Graphene Oxide and Tannins on Mechanical and Adhesive Properties
Source: Polymers (Basel). 2024 Apr 12;16(8):1081. doi: 10.3390/polym16081081 (PMC11055169; doi:10.3390/polym16081081)
Supplement: Supplementary file 1 [file polymers-16-01081-s001.zip › polymers-2935023-supplementary.pdf]

Supplementary Materials

Enhancing Alginate Hydrogels as Possible Wound Healing Patches: The Synergistic Impact of Reduced Graphene Oxide and Tannins on Mechanical and Adhesive Properties

Sebastián Carrasco [1], Luisbel González [1], Mauricio Tapia [1], Bruno F. Urbano [2], Claudio Aguayo [3], Katherina Fernández [1,*]

[1] Laboratorio de Biomateriales, Departamento de Ingeniería Química, Facultad de Ingeniería, Universidad de Concepción, Concepción, Chile

[2] Departamento de Polímeros, Facultad de Ciencias Químicas, Universidad de Concepción, Concepción, Chile

[3] Departamento de Bioquímica Clínica e Inmunología, Facultad de Farmacia, Universidad de Concepción, Concepción, Chile

* Correspondence: kfernandeze@udec.cl

1. Materials and Methods

1.1. Materials

Graphite powders (Flake, mesh 325) were bought from Asbury Online (Asbury Carbons, N.J., USA). Potassium permanganate powder ($KMnO_4$, 99.9%), sulfuric acid (H_2SO_4, 98%), phosphoric acid (H_3PO_4, 85%), hydrochloric acid (HCl, 37%), ferric chloride ($FeCl_3$-$6H_2O$≥99.0%) and Tris(hydroxymethyl)aminomethane were purchased from Merck (Darmstadt, Germany). Hydrogen peroxide (H_2O_2), sodium tetraborate decahydrate ($B_4Na_2O_7$-$10H_2O$ ≥ 99.5%) and glycerol were purchased from Furet (Concepción, Chile). Dopamine hydrochloride (DA-HCl ≥ 98.0%) and alginic acid sodium salt from brown algae with medium viscosity were purchased from Merck (Santiago, Chile). These chemicals were used as received without further purification. Milli-Q water was used throughout the study. For the sonication treatments, sonicator equipment (Elmasonic E60H model) was used for various sonication times as described below. All chemicals were of pro analysis quality or better.

1.2. Synthesis of Graphene Oxide (GO)

GO synthesis was performed by oxidation of natural graphite powder using the modified Hummers method [1]. 2.25 g of graphite were added to a mixture of 30 ml of H_3PO_4 and 270 ml of H_2SO_4. Once dissolved, 13.5 g of KMnO4 were slowly added and heated for 1 hour at 40°C. The mixture was then cooled to 25°C, and the oxidation was

stopped by adding H_2O_2 (60% v/v) until a color change from black to dark green was observed, without the presence of foam. Subsequently, it was centrifuged at 5000 rpm for 20 min and the precipitate was washed with HCl (10% v/v) until a light brown color was achieved. After washing with acid, three Milli-Q water washes were carried out, and it was centrifuged at 9000 rpm for 20 min. In this stage, the absence of chlorides was verified using $AgNO_3$. Then, a wash with ethanol and several washes with Milli-Q water were carried out to eliminate traces of alcohol. The solution was then dialyzed for 72 hours in 12 kDa dialysis bags and in 5L of Milli-Q water, with water changes every 24 hours. The dialyzed solution was placed in a cryogenic bath for 2 hours at -40°C and lyophilized for 72 hours.

1.3. Synthesis of reduced Graphene Oxide (rGO)

GO reduction was performed by the method proposed by Xu.17 A solution of 0.24 g of tris(hydroxymethyl)aminomethane in 200 mL of Milli-Q water was prepared, to which 0.05 g of DA and 0.1 g of GO were added. Once the GO dispersion was achieved, the pH was the sample was filtered through Whatman® No.1 filter paper and the retained solid was adjusted to 8.5 with HCl (5%) and sonicated for 20 min in an ice bath. Then, it was heated at 60 °C for 24 h with constant and gentle stirring to favor the reduction process. Subsequently, the sample was filtered through Whatman® No.1 filter paper and the retained solid was dialyzed in Milli-Q water for 72 h, with water changes every 24 hours. When dialysis was finished, the sample was filtered and dried at room temperature.

1.4. Pinus radiata Bark Extract Production

Pinus radiata bark extracts were produced through a pilot-scale extraction process. For this purpose, a reactor volume of 4 m3 and a vapor heating system composed of a shell and a tube heat exchanger with 6 m^2 heat transference area were used. In addition, a recirculation circuit for the extracted solution was implemented. Briefly, the Pinus radiata bark was ground with a double-knife mill to an average size lower than 20 mm. Then, the bark was dried at room temperature until a humidity of 24.5% (dry weight), and 100 kg (dry weight) of bark was soaked in an ethanol/water solution at a 1:20 ratio (w/v) for 120 min at 120 °C. Subsequently, the ethanol was evaporated under a vacuum (absolute pressure 0.05 bar) at room temperature. Thus, the water-insoluble particulate material after decanting and the water-soluble polyphenol fraction were obtained. Finally, the water-soluble polyphenols were lyophilized at room temperature and the obtained extracts were stored in sealed amber glass containers for further analysis.

2. Imagens

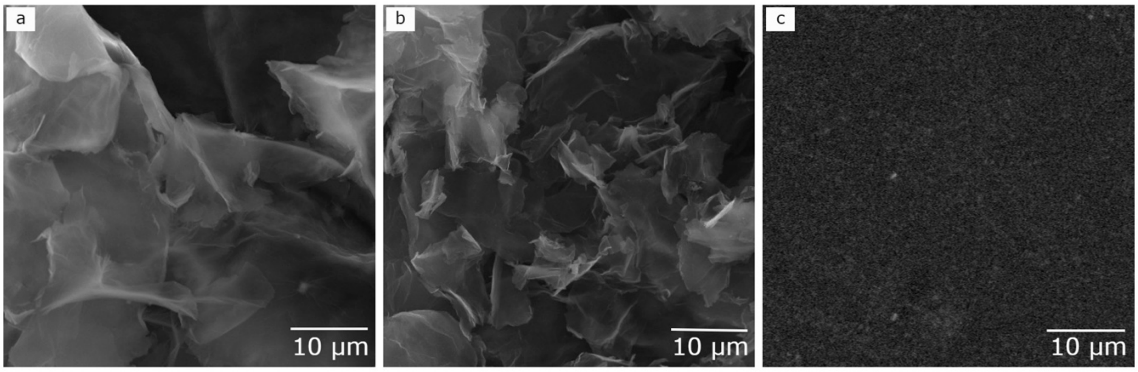

Figure S1. Scanning electron microscopy (SEM) images a) GO, b) rGO, c) Alg

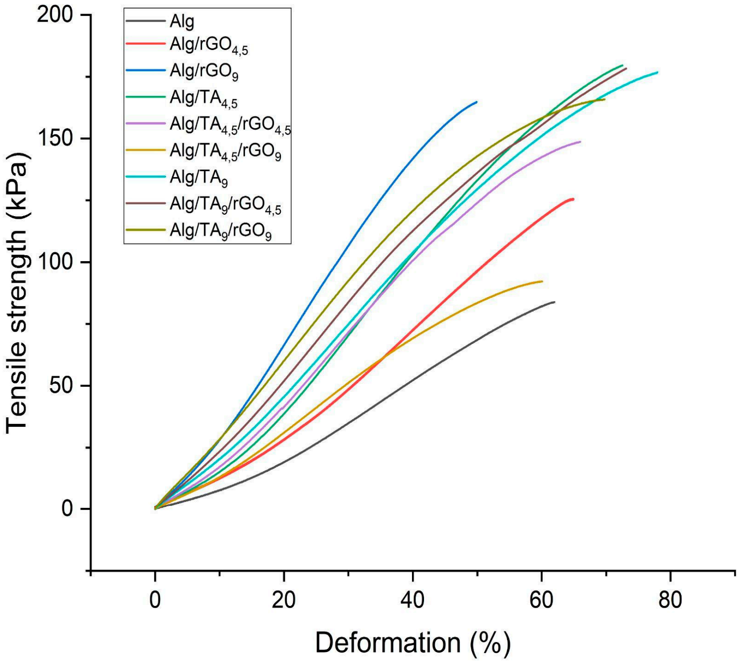

Figure S2. Tensile-deformation curves

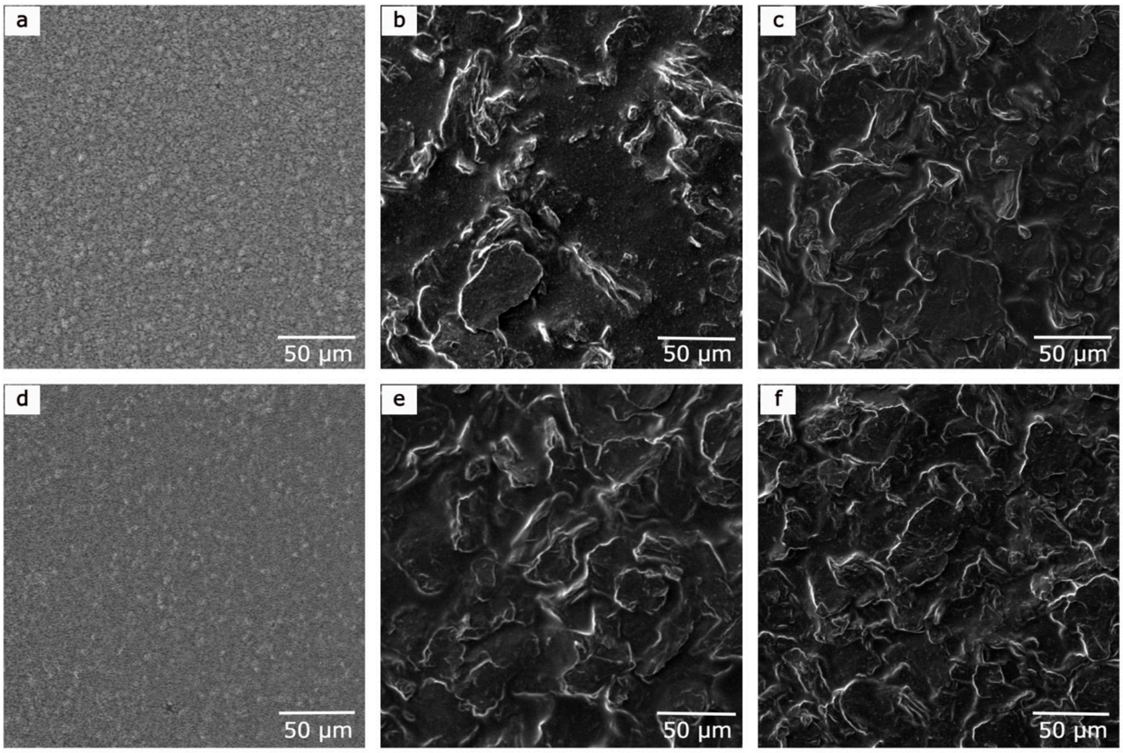

Figure S3. Scanning electron microscopy (SEM) images a) Alg/$TA_{4.5}$, b) Alg/$rGO_{4.5}$/$TA_{4.5}$, c) Alg/rGO_{9}/$TA_{4.5}$, d) Alg/TA_{9}, e) Alg/$rGO_{4.5}$/TA_{9}, f) Alg/rGO9/TA_{9}.

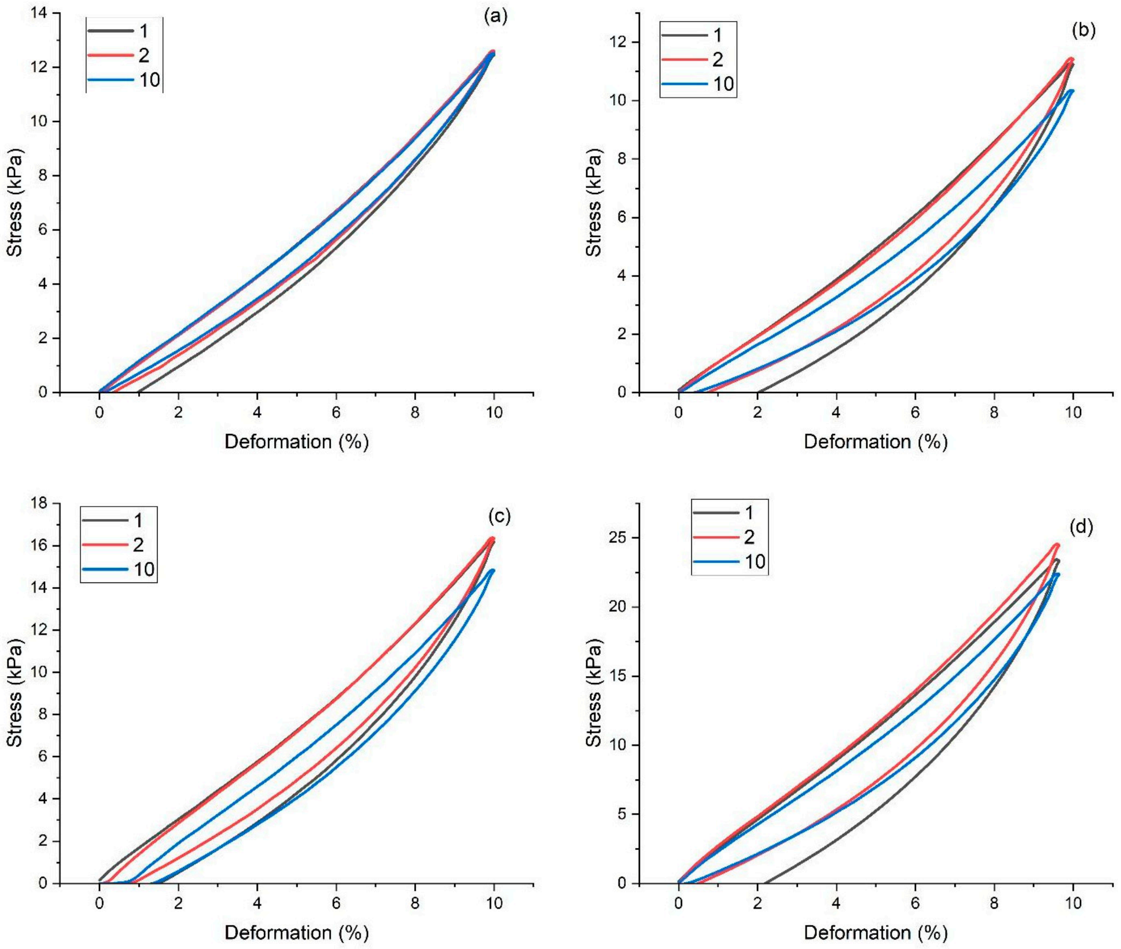

Figure S4. Cyclic tensile curves of (a) Alg, (b) Alg/rGO_9, (c) Alg/TA_9 and (d) Alg/$rGO_{4.5}$/TA_9.

Table S1. Average molecular weight number (Mn) of pine extracts at different percentages of sample development, determinate by GPC.

% sample development	Mn
10	438
25	749
50	1355
100	63277

Table S2. Interplanar distance of samples determined by XRD.

	2θ (°)	d (Å)
**GO**	8.65	10.22
**rGO**	21.72	4.09
**Alg**	21.20	4.19
**Alg/rGO$_9$**	21.50	4.13
**Alg/TA$_9$**	21.50	4.13
**Alg/rGO/TA$_9$**	21.70	4.10

References

[1] Hummers,W.S., Jr.; Offeman, R.E. Preparation of Graphitic Oxide. J. Am. Chem. Soc. 1958, 80, 1339.
